# Prag Letter

**Published:** 1882-08

**Authors:** Roswell Park

**Affiliations:** Prag


### ARTICLE XI.

PRAG, JULY 3, 1882.

EDITORS CHICAGO MEDICAL JOURNAL AND EXAMINER:

Prag is undoubtedly so little known to the majority of your readers as a great medical center, that it deserves all the space I can devote to it in this letter. The University of Prag was founded by Charles IV in 1348, and is consequently the oldest in all Germany. His successor, Wenzel, desiring to limit the privileges of foreign students, many hundreds of them deserted Prag, and founded the University of Leipzig sixty-one years later. Beside being the oldest, it was for centuries the best attended center of learning in all Europe. During this time it had six thousand students and upwards, two thousand of these being in the medical department. At present it has, all told, about two thousand, four hundred of these being students of medicine. More than half of these are of Bohemian birth, and many speak no German at all. For these lectures in Czeckisch are given, there being really two series of parallel lectures, one in German and the other in the vernacular.

The medical department has numbered in its faculty some of the best men that Germany and Austria have ever produced.

To speak only of the last few years, Klebs, the celebrated pathologist, has but recently left Prag for Zurich; Toldt, the present professor of anatomy, has no superiors, and the present century lost one of its brightest surgical ornaments when the lamented von Heine died, three years ago. He was succeeded by Gussenbauer, of whom I shall have much to say. Ranking with Czerny as one of Billroth's most favorite and brilliant scholars, after being private docent in Vienna for a time, he was called to the chair of surgery in Liège (Belgium); from here he was nominated as Heine's successor. A most industrious student and hard worker; a most fascinating clinical teacher; a fearless operator, I esteem him as already the equal of his renowned preceptor, and I dare say that he will succeed Billroth at Vienna when the time comes.

The hospital here, that is, the main part of it, is about a hundred years old; nevertheless, everything is kept clean and neat. There are also a children's hospital, a foundling asylum, and a military hospital on adjoining lots, and close by are the insane asylum, with sixteen hundred patients, and the new maternity hospital. This latter is the most elegant and best adapted building of its kind I have yet seen on the continent. It is on the pavilion plan, of red brick, thoroughly modern in its external features and internal appointments, and architecturally a thing of beauty. It accommodates nearly three hundred patients. Breisky, who is well known to American obstetricians and gynæcologists, at least, is its shining light. Here the advantages are as good as in Vienna, except that they have perhaps only two-fifths as many births during the year; but they have at least three thousand, an ample number for clinical purposes. Here a student, by payment of five dollars, can have a bed in the students' quarters, and be called to all the labor cases on his side, for six months.

So that, excluding the insane asylum, there are at least a thousand beds to which students have access, and a like number of patients available for clinical purposes. Besides these, there are a large number of out-patients flocking every day to the general hospital.

Gussenbauer has in his service about a hundred and twenty-five in-patients, and an unknown number of out-patients. His clinic lasts nearly two hours every day, and consists nearly wholly of rare and interesting cases. For instance, the first week I was here there were four exsections of the upper jaw, one or two of the lower, several amputations, and numerous other difficult operations. The number of patients with more or less malignant tumors is astonishing. Every day three or four are presented, and the half are not seen in public; but having "the run" of the wards, I cannot help but notice them.

The other day Gussenbauer attacked what proved to be the most complicated case of ovarian tumor I have yet seen. There were adhesions everywhere and to everything; to the abdominal walls; to the viscera and omentum; to the vena cava; to the ureters; and a smaller cyst dipping down in the pelvis was adherent to bladder, rectum and pelvic fascia. After infinite pains, he removed the whole mass, the operation lasting nearly two hours, and up to date the patient has not had a bad symptom, and bids fair to recover without one.

The previous day he extirpated four inches and a half of cancerous rectum, with the large portion of the prostate. The patient did well for two days, and then rapidly sank. Autopsy showed that he died of fatty heart and thrombosis, but there was not the slightest evidence of peritonitis or sepsis.

And to-day Gussenbauer made the most difficult operation I have yet seen him do; a resection of the pylorus. The patient was a middle-aged woman, who had had symptoms of stomach trouble for three years. A diagnosis of cancer was made three months ago, and last week she was sent in for operation. The tumor being freely movable, it seemed an excellent case for resection.

Bichloride of methylene was used, as it is here in all laparotomies, because it so seldom causes nausea. It is administered with a Clover apparatus. Thymol was used in the spray. The preliminary incision was made in the middle line, both as being more indicated in this case, and as leading to less hæmorrhage. Usually the incision is made obliquely. The diseased part, being brought to light, was carefully deprived of its omentum, on both sides, numerous catgut ligatures being required. The diseased part having been thus excised, the slow and tedious process of bringing the parts into apposition and properly suturing them was begun.

Either such a long description or one or two diagrams being required to explain this whole process, I must defer a detailed account, perhaps until another occasion. This part of the operation being happily completed, the parts were thoroughly cleansed and returned, and the external wound completely closed. The operation lasted three hours, but such thorough care was taken to prevent hæmorrhage, that I doubt if two ounces of blood were lost. The patient suffered from shock, but was put to bed in as good shape as could be expected. I should have said that two or three suspicious glands in the greater omentum were also removed.

This is Gussenbauer's second case of the kind; his first having been unsuccessful. Although not the first to practice it on the human being, he was among the first to investigate the possibilities of the operation on the dog and other animals.

It may be worth while to state that he uses chloroform invariably in all his operations, with the exception above noted; and I have been both surprised and pleased with the ease with which they anæsthetize patients and keep them under its influ ence; pleased because I am in favor of chloroform, and have always written and talked in favor of it.

Altogether, I think I have said enough to show the vast and varied amount of material in this one clinic, and to render my homage of admiration for its chief. I may add that Prof. Weiss (formerly in Paris) has the other surgical clinic, which is said to be equally rich in material. His lectures are in Bohemian.

Let me devote a little space to Hasuer, the oculist. His name is familiar only to the students of ophthalmological literature, at home, and yet I suppose he is the most expert and rapid operator, perhaps of the present century. The 16th of May is the day allotted in the calendar to St. John Nepomuck, the patron saint of Bohemia. On this day the halt, the maim and the blind come in from all the surrounding country for treatment. Among this motley crowd are a great many cataract cases, and on one of these occasions, having his patients all made ready, Hasuer is known to have operated on *twenty-three eyes in one hour*. I have seen him several times, and can testify to the wonderful rapidity and certainty of his movements. I saw him one day remove two cataracts from different patients in different rooms, and also operate on both sides for strabismus, *within six minutes.* Of course no anæsthetic was used, and all the dressing was left to the assistants. Hasuer has his own method of extracting cataract. He uses a Beers' knife, makes an incision closely corresponding to the Græfe cut, and then, instead of an *iridectomy*, he performs an *iridotomy.* Most of his results are wonderfully good. Surely the method is deserving a trial on your side of the ocean.

Traufal is not as well known at home as his Vienna colleagues, Politzer and Gruber, but is most favorably known over here. In his clinics are found more than the usual number of cases that repay close study.

I doubt if in any one of the Vienna clinics a larger number of eye or ear cases can be found than in those here; certainly Hasuer's annual showing of nearly two hundred and fifty cataract extractions, will compare very favorably with either Arlt's or Jaeger's.

The skin clinic here is also interesting, though in this respect Prag can hardly compare with Vienna.

If one desires to study either microscopic or gross, normal or pathological anatomy, the opportunities abound and are excellent. The interest in pathological histology which Klebs aroused will not subside under Chiari, who has recently been called here from Vienna. Every morning from five to ten sections are made, and cadavers can be secured for purposes of dissecting or practicing operations for from forty to seventy-five cents.

In every respect, then, Prag deserves the attention of any one going abroad to study. Everything is cheap, even whatever private instruction a man may want. I think no one ought to study in Europe until he has done some work and some practice at home; but to such I can most cordially commend Prag, both as a place where he will be treated much better in every respect than in Vienna, and as a field for study which compares most favorably with Berlin and Vienna, and perhaps ranks next to them.

ROSWELL PARK.

